# Nilotinib (Tasigna™) in the treatment of early diffuse systemic sclerosis: an open-label, pilot clinical trial

**DOI:** 10.1186/s13075-015-0721-3

**Published:** 2015-08-18

**Authors:** Jessica K. Gordon, Viktor Martyanov, Cynthia Magro, Horatio F. Wildman, Tammara A. Wood, Wei-Ti Huang, Mary K. Crow, Michael L. Whitfield, Robert F. Spiera

**Affiliations:** Department of Rheumatology, Hospital for Special Surgery, 535 East 70th St, New York, NY 10021 USA; Geisel School of Medicine at Dartmouth, Department of Genetics, Remsen 7400, Hanover, NH 03755 USA; Weill Cornell Medical Center, Department of Dermatopathology, 525 East 68th St, New York, NY 10065 USA; Weill Cornell Medical Center, Department of Dermatology, 1305 York Ave, New York, NY 10021 USA

## Abstract

**Introduction:**

Tyrosine kinase inhibitors (TKI) are medications of interest in the treatment of Systemic Sclerosis (SSc) because of their ability to inhibit pathways involved in fibrosis. In this open-label pilot trial, our objectives were to assess the safety, efficacy, and molecular change associated with treatment of patients with diffuse cutaneous (dc)SSc with the TKI nilotinib (Tasigna™).

**Methods:**

Ten adult patients with early dcSSc were treated with nilotinib. Primary endpoints were safety and change in modified Rodnan Skin Score (MRSS) after 6 months. Lesional skin biopsies at baseline, 6 and 12 months of treatment were assessed by histopathology, immunohistochemistry, and DNA microarray.

**Results:**

Patients had early and active dcSSc with median disease duration of 0.7 years (range 0.5, 1.7) and increasing MRSS in the month prior to baseline (mean +2.9, p=0.02). Seven out of ten patients completed 6 and 12 months of treatment. Seventy-one adverse events (AEs) including 2 serious AEs were observed, and 92 % of AEs were grade 1-2. Two patients discontinued the medication due to mild QTc prolongation. MRSS improved by a mean of 4.2 points (16 %) at 6 months and by 6.3 points (23 %) at 12 months in the 7 completers, p=0.02 and 0.01, respectively. Patients with a decrease in MRSS >20 % from baseline at 12 months (classified as improvers) had significantly higher expression of transforming growth factor beta receptor (*TGFBR*) and platelet-derived growth factor receptor beta (*PDGFRB*) signaling genes at baseline than non-improvers, and the expression of these genes significantly decreased in improvers post-treatment.

**Conclusion:**

Nilotinib was well tolerated by the majority of patients in this study, with tolerability limited primarily by mild QTc-prolongation. Significant MRSS improvement was observed in these early, active patients, but is not conclusive of treatment effect given the open-label study-design and small number of patients in this pilot study. Improvers had higher levels of expression of genes associated with *TGFBR* and *PDGFRB* signaling at baseline, and a significant decrease in the expression of these genes occurred only in patients with higher MRSS improvement. The findings of this pilot study warrant more conclusive evaluation.

**Trial registration:**

Clinicaltrials.gov NCT01166139, July 1, 2010.

**Electronic supplementary material:**

The online version of this article (doi:10.1186/s13075-015-0721-3) contains supplementary material, which is available to authorized users.

## Introduction

Systemic sclerosis (SSc; scleroderma) is a multisystem disorder characterized by vasculopathy, autoimmunity, inflammation, and fibrosis [1]. Patients with diffuse cutaneous SSc (dcSSc) have increased morbidity and mortality when compared to patients with other rheumatic diseases [2]. Although several medications are used to treat the skin disease associated with dcSSc, there are no universally effective therapies, and the treatment of scleroderma skin disease remains an area of unmet need [3].

Transforming growth factor beta (*TGFB*) and the platelet-derived growth factor (*PDGF*) are cytokines implicated in the pathological fibrosis of dcSSc [4, 5]. Nilotinib (Tasigna™; Novartis, Basel, Switzerland) is a tyrosine kinase inhibitor (TKI) with antagonistic activity against Abelson tyrosine kinase (*c-Abl*), the *PDGF* receptor (*PDGFR*), and other tyrosine kinases. It is approved in the USA for the treatment of chronic myelogenous leukemia (CML) [6]. It is a therapy of interest for dcSSc because of its ability to interfere with both *TGFB* and *PDGF* signaling. Nilotinib has been shown to decrease fibrosis in vitro and in bleomycin models of SSc similarly to imatinib [7]. However, these models have shortcomings in their ability to predict clinical impact in SSc [8].

Several groups have studied imatinib for the treatment of dcSSc with variable experiences [9]. Unfortunately, none of the studies have been definitive due to the open-label study design, inclusion of patients with limited cutaneous SSc (lcSSc) and morphea, or inadequate power [10–13]. Adverse events (AE), in particular fluid retention, were prominent in these studies, but may be less frequent when imatinib is used at a low dose [14]. Although fluid retention has been observed in other populations, it has been particularly problematic in dcSSc, even leading to the early termination of one trial. Subcutaneous edema may also elevate the modified Rodnan skin score (MRSS), as edema can be difficult to distinguish from dermal thickening.

Nilotinib is a second generation TKI that blocks *c-abl* and *PDGFR* (more potently *c-abl* than imatinib and less potently *PDGFR*) [15]. In populations with CML, edema has been seen in only 10 % of patients, and this represents an advantage of nilotinib over imatinib as a candidate therapy for SSc. We report here the results of our pilot trial.

## Methods

### Study subjects

Patients were recruited from November 2010 until December 2011. Patients fulfilled the 1980 American College of Rheumatology classification criteria for SSc [16] and had the diffuse subtype [17]. Patients were over 18 years old, had a disease duration <3 years since the first SSc-related symptom other than Raynaud’s phenomenon, and had a baseline MRSS ≥16. Patients were excluded if they had a baseline corrected QT (QTc) interval on electrocardiogram (EKG) >450 msec. Additional exclusion criteria included treatment with immunosuppressive therapies within 3 months before baseline (including prednisone equivalent >10 mg), pregnancy, serious medical conditions, diffusion capacity of carbon monoxide (DLCO) <30 % predicted, or ejection fraction (EF) <50 %.

### Study design

This was an investigator-initiated, single-center, open-label pilot study. The primary objective was to assess the safety and tolerability of nilotinib in patients with dcSSc as assessed by the number of AE and serious adverse events (SAE). The primary efficacy endpoint was change in MRSS after 6 months of treatment. Secondary efficacy endpoints included change in MRSS at 12 months, forced vital capacity (FVC) and DLCO on pulmonary function testing (PFT) as well as change in the short form 36 (SF-36) mental (MC) and physical components (PC) and scleroderma health assessment questionnaire disability index (SHAQ-DI). Skin biopsies were assessed using histopathologic and immunohistochemical analysis, and gene expression profiling with DNA microarray to assess change with treatment and explore the biologic basis of the clinical changes observed.

The protocol was approved by the institutional review board at the Hospital for Special Surgery. Patients provided written informed consent before enrollment. An independent Data and Safety Monitoring Board (DSMB) regularly reviewed safety data. The trial was registered at ClinicalTrials.gov (NCT01166139).

Patients were assessed at visits occurring every month for AE ascertainment, interval history, physical examination, clinical laboratory measurements, and 12-lead EKG, and called our center about issues between visits. AE were listed according to the common terminology of the National Cancer Institute [18]. AE were graded as follows: 1 - mild, not requiring intervention; 2 - moderate, requiring minimal local or noninvasive intervention; 3 - severe, 4 - life-threatening; 5 - death. AE were attributed as unrelated, unlikely related, possibly related, probably related, or definitely related to the study medication in the opinion of the investigators and as adjudicated by the Data Safety Monitoring Board (DSMB). The MRSS was measured at screening, baseline, and every 3 months by the same physician (RS or JG). PFT with measurement of FVC and DLCO was performed at baseline and after 6 and 12 months.

### Dosing

Patients started nilotinib 200 mg by mouth daily for 1 week, which was up-titrated to 200 mg twice daily for 3 weeks and then to 400 mg twice daily. A 12-lead EKG with measurement of QTc was checked 1 week after any dosing change. This titration scheme is not required but was chosen by the investigators to carefully observe any dose-related side effects [19].

### Dermatopathology

Two 3-mm punch biopsies of lesional, extensor-surface, forearm skin were performed at baseline, and after 6 and 12 months of treatment. The post-treatment biopsies were taken 1 cm adjacent to the previous biopsy. At each time point one specimen was formalin-fixed and paraffin-embedded, and the other was stored in RNAlater (Thermo Fisher Scientific, Waltham, MA, USA). Sections for histopathology were stained with hematoxylin and eosin (H&E), anti-α-smooth muscle actin (αSMA), and Masson trichrome (TC), CD-34, pro-Collagen (proCol), phosphorylated cAbl (p-cAbl), and phosphorylated PDGFR (p-PDGFR) using standard techniques. A dermatopathologist (CM), blinded to treatment status, compared each case. Slides were scored semiquantitatively using a scale (0, 1, 2, 3) based on collagen density and degree of infiltrate on H&E, and the intensity of immunohistochemical stains. Skin thickness was measured by a micrometer from the epidermis to the subcutis. Eccrine coils and hair follicles were counted per section.

### Gene expression by DNA microarray

Tissue samples stored in RNAlater were homogenized and RNA was purified as previously described [20]. RNA integrity was assessed using the Agilent 2100 Bioanalyzer (Agilent Technologies, Santa Clara, CA, USA) and all samples had RNA integrity numbers (RIN). Total RNA (25 ng) was amplified and labeled with Agilent Low Input Quick Amp Labeling Kit. cRNA was hybridized to Agilent SurePrint G3 Human Gene Expression 8x60K Microarrays (G4851A). Agilent Feature Extraction Image Analysis Software (Version 10.7.3) was used to extract data from raw microarray image files. Microarray data were log2-lowess normalized and filtered for probes with intensity ≥1.5-fold over local background in Cy3 or Cy5 channels. Expression values were multiplied by −1 to convert them to log2(Cy3/Cy5) ratios. Probes with >20 % missing data were excluded resulting in 31,762 probes that passed the filtering criteria. Paired samples (baseline and post-treatment biopsies at 12 months) were available for six patients and were used for analyses. We categorized patients as improvers based on a decrease in MRSS of >20 % at 12 months compared to baseline, and 4/6 patients with paired 12-month biopsy specimens met this criterion. The expression data from the study are deposited to NCBI GEO [GEO:GSE65405].

### Microarray data pre-processing

Microarray data were pre-processed and analyzed using GenePattern modules [21] with default parameters unless stated otherwise. Expression data in Stanford preclustering (PCL) file format were converted to Gene Cluster Text (GCT) file format via PclToGct module. Missing expression values were imputed via ImputeMissingValuesKNN module. In total, 31,762 probes were collapsed to 18,398 unique gene symbols via CollapseDataset module using annotation file for the Agilent 8x60K microarray platform. Class label (CLS) files were created via ClsFileCreator module to define phenotypic classes for microarray samples. Expression data were median-centered gene-wise in Cluster 3.0 [22].

### Differential expression analysis

For both baseline (improvers vs non-improvers) and improver (baseline vs post treatment) comparisons, the ComparativeMarkerSelection [23] module from GenePattern was used to identify differentially expressed genes. The ‘number of permutations’ parameter was set to 0 and the ‘log transformed data’ parameter was set to ‘yes’. For improver comparison, the ‘test statistic’ parameter was set to ‘Paired *T*-Test’. Expression data for significant genes (*p* <0.05, not corrected for multiple hypothesis testing) were extracted via the ExtractComparativeMarkerResults module and converted to PCL file format using the GctToPcl module. Expression data were then hierarchically clustered gene-wise and array-wise in Cluster 3.0 using the uncentered correlation similarity metric and average linkage clustering method, and were visualized in TreeView [24].

### Pathway enrichment analysis

For baseline and improver comparisons, pathways with significant changes in expression were identified by gene set enrichment analysis (GSEA) [25, 26] and single-sample GSEA (ssGSEA) [27] using corresponding GenePattern modules. All GSEA analyses were corrected for multiple hypothesis testing. GSEA and ssGSEA were run against the Canonical Pathways database comprising gene sets from several pathway databases. For GSEA, the ‘permutation type’ parameter was set to ‘gene set’. ssGSEA enrichment scores were normalized by dividing by the maximum ssGSEA enrichment score for this expression dataset. Normalized ssGSEA enrichment scores for significant pathways (false discovery rate (FDR) <5 %) were extracted, clustered and visualized as described above for the expression data.

### Intrinsic subset assignment

Intrinsic probes (n = 995) from Milano et al. [28] were collapsed to 793 unique genes. Separately the entire nilotinib dataset comprising expression data for 24 samples (including all baseline, 6-month and 12-month biopsies) was combined with 4 healthy control samples analyzed on the same DNA microarray platform to provide the proper data distribution across groups. From these samples 27,276 probes passed quality filters and were collapsed to 16,580 unique genes. Overlap with the 793 unique genes from Milano et al. resulted in 651/793 genes (82.1 %) in common between the two datasets. These 651 genes were used to organize the gene expression data from nilotinib and healthy control samples by unsupervised hierarchical clustering.

Intrinsic subset assignment was performed using the 651 intrinsic genes to calculate Spearman non-parametric statistics (correlation coefficients and *p* values) between each sample from the study and three centroids corresponding to fibroproliferative, inflammatory and normal-like samples from Milano et al. Limited was excluded because no limited SSc samples were included in this study. Centroids were created by averaging expression values for each gene across all samples assigned to a given intrinsic subset in Milano et al. The intrinsic subset assignment for each nilotinib sample was to the subset centroid with the highest Spearman correlation coefficient and the lowest *p* value.

### Statistical analysis

Descriptive analysis of AE and SAE was performed for assessment of clinical data. Continuous clinical variables were analyzed using the unpaired (baseline vs baseline) and paired (baseline vs post treatment) *t* test.

For gene expression analysis, the relationship between baseline intrinsic subset and response status was analyzed using the chi-square test. In cases where three or more groups were compared, one-way analysis of variance (ANOVA) was used and *p* values were corrected for multiple testing using Tukey’s multiple comparisons test with a single pooled variance. Statistical analyses were performed using SPSS software version 17.0 (IBM, Armonk, NY, USA) and GraphPad Prism Windows 6.05 (GraphPad Software, San Diego, CA, USA).

## Results

### Patients

Nineteen patients were screened for study inclusion. Five were excluded due to baseline QTc >450 msec and four were excluded due to meeting other exclusion criteria. Baseline characteristics of all patients are listed in Table 1. The patients in this study represented a group of patients with early and active dcSSc. The median disease duration based on the time since the first non-Raynaud’s symptom of SSc was 0.7 years (range 0.5−1.7). In the 1-month screening period prior to starting medication, 70 % patients had an increasing MRSS, and the group had a mean MRSS increase of 2.9 points, *p* = 0.02. Fifty percent of patients were positive for the RNA Polymerase 3 antibody. Seven patients tolerated 6 months of nilotinib and continued treatment for at least 12 months. Two patients had to discontinue nilotinib within the first month due to grade 1 (QTc of 453 msec) or 2 (QTc of 483 msec) QTc prolongation. One patient discontinued due to progression of preexisting coronary artery disease after 3 months of treatment.

**Table 1:** Baseline characteristics

Variable	Value
Age, years, median (range)	46 (18−69)
Sex, female, n (%)	8 (80 %)
Race, n (%)	
Caucasian	5 (50 %)
African American	3 (30 %)
Disease duration, median (range)	0.7 (0.5−1.7)
ANA-positive, n (%)	9 (90 %)
Anti-Scl 70-positive, n (%)	3 (30 %)
RNA polymerase III-positive, n (%)	5 (50 %)
MRSS at baseline, mean ± SD	30.1 ± 8.2
Mean change in MRSS in 1 month prior to baseline, mean ± SD	+2.9 ± 3.4
Tendon friction rubs, n (%)	4 (40 %)
Previous treatment, n (%)	
Methotrexate	2 (20 %)
No immunosuppression	8 (80 %)

*ANA* antinuclear antibody, *MRSS* modified Rodnan skin score

### Adverse events

Seventy-one AE including two SAE were observed during the 12-month treatment period (Additional file 1); 75 % were considered to be at least possibly related to nilotinib (Table 2). Of the AE, 92 % were grade 1 or 2 and no unexpected events were observed. One common AE was grade 1 (<2.5 × upper limit of normal) liver function test (LFT) abnormality, which occurred in 50 % of patients. All LFT abnormalities are shown in Additional file 1, and all were asymptomatic. No medication adjustment was necessary and the abnormal values resolved when the laboratories were rechecked. There were no LFT abnormalities of grade 2 or higher. QTc prolongation occurred in 60 % of patients. Although QTc prolongation was grade 1 or 2, our protocol required discontinuation of two patients due to this. The other four patients had normalized QTc on repeat EKG or adjustment of medication dosage.

**Table 2:** Adverse events

Adverse event	Number of patients reporting adverse events	Grade
Blood and lymphatic system disorders		
Anemia	3	1
Decreased WBC count	1	1
Cardiac disorders		
Prolonged QTc	6	1, 2
Coronary artery disease	1	4
Syncope	1	4
Endocrine disorders		
Hyperglycemia	3	1
Gastrointestinal disorders		
Nausea	2	1
Diarrhea	1	1
Reflux	1	1
General disorders		
Headache	3	1
Hepatobiliary disorders		
Elevated total bilirubin	5	1
Increased AST	5	1
Increased ALT	4	1
Increased amylase	2	1
Increased lipase	2	1
Metabolism and nutrition disorders		
Low inorganic phosphorus	1	1
Vitamin D deficiency	1	1
Nervous system disorders		
Dizziness	1	1
Psychiatric disorders		
Anxiety	1	1
Concentration impairment	1	1
Reproductive system and breast disorders		
Erectile dysfunction	1	1
Skin and subcutaneous tissue disorders		
Rash acneiform	1	2
Alopecia	1	1

All AEs considered at least possibly related to nilotinib are listed in order of frequency and by Common Terminology Criteria for Adverse Events (CTCAE) grade. *WBC* white blood cells, *QTc* corrected QT interval, *AST* aspartate aminotransferase, *ALT* alanine aminotransferase

Two SAEs occurred both of which were considered possibly related to the medication. One patient experienced a syncopal episode and was hospitalized. The medication was held and the patient’s evaluation, including EKG with QTc measurement and telemetry, was normal with the syncopal episode considered to be a vasovagal episode. However, when the patient followed up and off medication for 3 weeks, her baseline was QTc >450 msec and she could not restart medication as she now met an exclusion criterion. Another patient with known prior history of coronary artery disease (CAD) was hospitalized for coronary artery bypass graft (CABG) surgery after 3 months of treatment. The patient had multiple risk factors for vascular disease including age, diabetes mellitus, longstanding essential hypertension, hyperlipidemia, and obesity. She had atypical chest pain, which she described as heartburn and which had been attributed to her gastroesophageal reflux disease predating the trial. She underwent cardiac testing during the trial, which in turn led to indication for CABG. It is possible that worsening of her vascular disease was related to nilotinib use, although with her multiple risk factors and symptoms that predated the use of nilotinib, the attribution of this SAE was not clear cut.

### Efficacy

#### Skin

In the seven patients who completed 12 months of treatment, the MRSS statistically significantly improved from a mean of 26.9 ± 5.4 to 22.7 ± 8 (by 16 %) at 6 months and to 20.6 ± 7.7 (by 23 %) at 12 months, *p* = 0.02 and 0.0112, respectively (Table 3). The mean change in the MRSS in the completers with the RNA Polymerase3 antibody (n = 4) was −7.0 ± 1.8 versus −5.5 ± 7.5 in those without (n = 3). This was not statistically significant, *p* = 0.68.

**Table 3:** Clinical outcomes in completers (n = 7)

Measurement	Baseline	6 Months	12 Months	*P* value 6 Months	*P* value 12 Months
MRSS	26.9 ± 5.4	22.7 ± 8	20.6 ± 7.7	0.02	0.01
FVC	77.4 ± 12.9	75.4 ± 12.6	71.7 ± 11.7	0.17	0.054
DLCO	72.0 ± 9.9	69.9 ± 18.1	69.3 ± 12.4	0.56	0.25
PGA	62.5 ± 10.8	40.2 ± 12.7	32.3 ±15.3	<0.01	<0.01
SHAQ	0.71 ± 0.34	0.79 ± 0.59	0.86 ± 0.66	0.86	0.74
ESR	17.2 ± 14.5	23.0 ± 22.1	22.6 ±17.7	0.19	0.1506
SF-36 MC	51.5 ± 8.2	52.8 ± 7.6	52.1 ± 5.6	0.74	0.90
SF-36 PC	43.1 ± 9.5	40.3 ± 9.5	41.7 ± 9.1	0.37	0.74

*MRSS* modified Rodnan skin score, *FVC* forced vital capacity, *DLCO* diffusion lung capacity of carbon monoxide, *PGA* physician global assessment, *SHAQ* scleroderma health assessment questionnaire, *ESR* erythrocyte sedimentation rate, *SF-36 MC* short form-36 health survey mental component, *SF-36 PC* short form-36 health survey physical component

Two patients who discontinued study medication had follow-up MRSS at 6 months. One patient’s MRSS decreased from 43 to 21 on 4 months of mycophenolate, after stopping nilotinib after less than 1 month due to QTc prolongation. One patient’s MRSS was relatively unchanged from 44 to 46 after stopping nilotinib after 3 months. The third patient continued her care locally.

#### Pulmonary efficacy

Out of 10 patients, 3 had ILD at baseline and 2 of those patients did not complete the study. The mean FVC was 77.4 ± 12.9 % predicted at baseline, 75.4 ± 12.6 % at 6 months and 71.7 ± 11.7 at 12 months, *p* = 0.17 and 0.054, respectively. The DLCO was 72.0 ± 9.9 % predicted at baseline and 69.9 ± 18.1 % at 6 months, and 69.3 ± 12.4 % at 12 months, *p* = 0.56 and 0.25, respectively. The completer with ILD had stable PFT parameters. The trend to decline in FVC was driven by one patient without evidence of ILD at entry, who went on to develop mild reticular opacities after one year.

#### Other assessments

The physician global assessment (PGA) significantly improved from 62.5 ± 10.8 at baseline to 40.2 ± 12.7 at 6 months, and to 32.3 ±15.3 at 12 months, *p* = 0.0033 and 0.0013, respectively. There were no significant differences in ESR, SHAQ-DI, SF-36 mental or physical components, oral aperture, hand extension, or finger-to-palm distance.

#### Dermatopathologic assessment

**Fig. 1:**
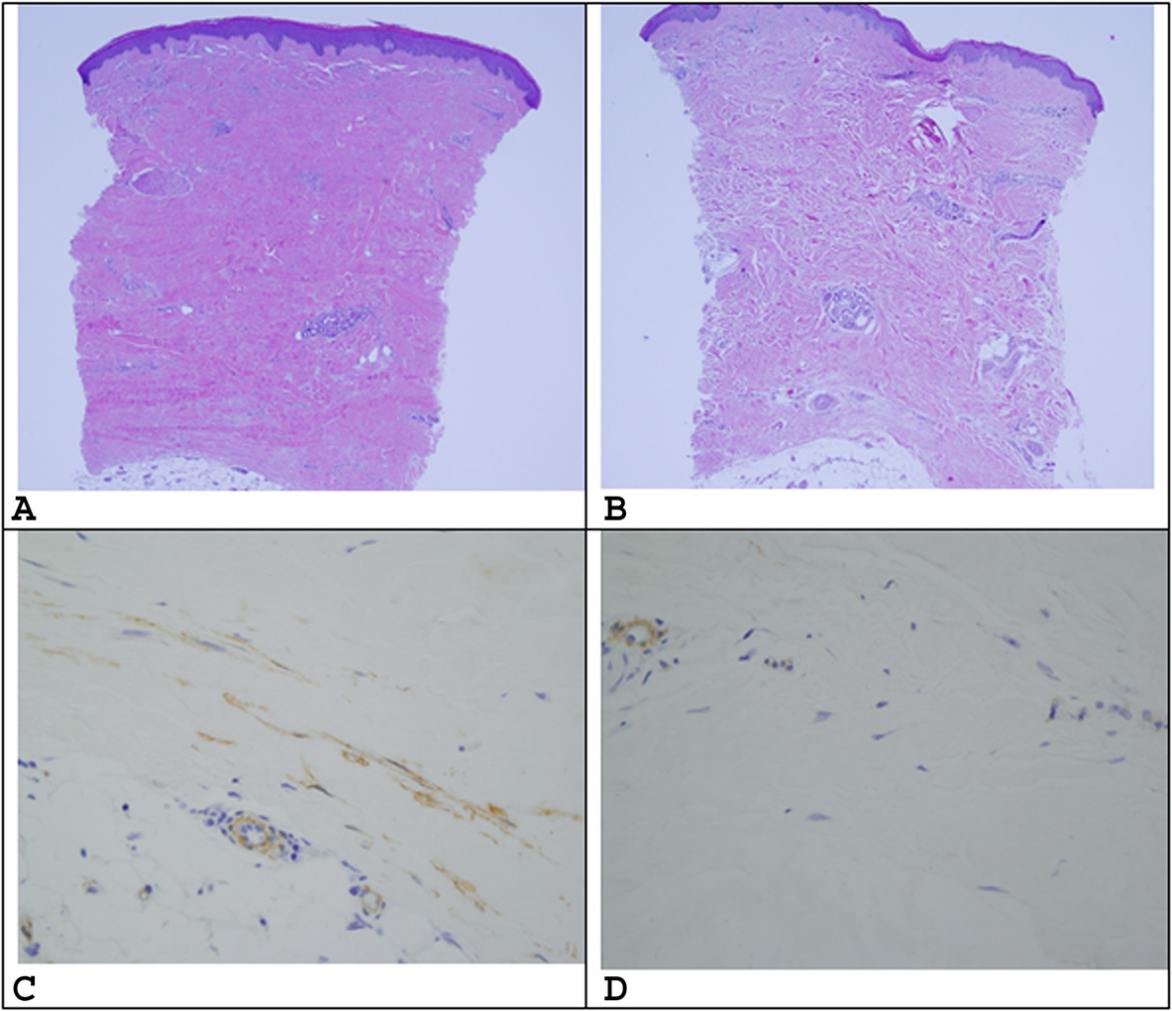
Photomicrographs of forearm skin biopsy from one patient as studied by H&E at 0 (**a**) and 12 (**b**) months and by alpha smooth muscle actin (α-SMA) at 0 (**c**) and 12 (**d**) months of treatment. This specimen demonstrates morphological improvement as evidenced by decreased thickness of collagen bundles and increased interstitial space between the collagen bundles. Decreased intensity α-SMA is seen in this specimen as well. However, when looking at all of the specimens overall, significant morphological change was not observed

Eight patients had biopsies at baseline and 6 months and six patients had biopsies at 12 months. The mean skin thickness at baseline was 2.3 ± 0.6 mm, 2.7 ± 0.6 mm at 6 months, and 2.6 ± 0.3 mm at 12 months, with no statistically significant difference (*p* = 0.06 at 6 months and *p* = 0.36 at 12 months). One patient sample is shown in Fig. 1. There was no significant change in the group in collagen density, degree of infiltrate on H&E, number of follicles and eccrine structures, and staining intensity of α-SMA, trichrome, CD-34, pro-Collagen, p-PDGFR, or p-cAbl; these data are summarized in Additional file 2. There was no difference in qualitatively assessed staining of pPDGFR or p-cAbl staining between those patients with MRSS improvement >20 % and those who did not meet this level of improvement.

### Gene expression changes in skin

#### Intrinsic gene expression subset assignment before and after treatment

**Fig. 2:**
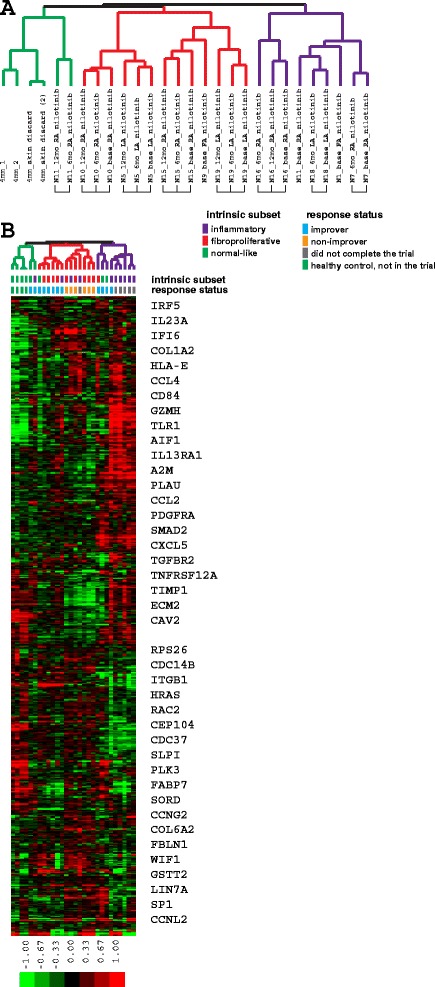
Intrinsic subset assignment. **a** Nilotinib sample tree: *green* normal-like, *red* fibroproliferative, *purple* inflammatory intrinsic subset samples, based on the expression patterns of intrinsic genes. Square brackets indicate samples from the same patient. **b** Heat map of intrinsic genes. The *intrinsic subset* row refers to subset assignments based on Spearman correlation statistics

We used intrinsic subset genes from Milano et al. [28] (see Methods and Additional file 3) to assign the samples in this trial to intrinsic gene expression subsets (Fig. 2a). Subsets were assigned based on Spearman correlation statistics to a gene expression centroid calculated from Milano et al. samples (see Methods and Additional file 4). Clustering was primarily driven by a strong inflammatory signature evident in the skin of these patients (Fig. 2b). Baseline intrinsic subset assignment showed that three out of four improvers were classified as non-fibroproliferative at baseline (two inflammatory and one normal-like), whereas one improver was classified as fibroproliferative. The patients who improved generally lost their major subset signature and three out of four were classified as normal-like post treatment (Table 4). The two non-improvers were assigned to the fibroproliferative subset (*p* = 0.0833, chi-square test for enrichment of fibroproliferative patients as non-improvers) and remained in the fibroproliferative subset post treatment, consistent with the results of Pendergrass et al. [29]. Slight discrepancies between the Spearman correlation intrinsic subset assignments and the hierarchical clustering tree occured mainly in post-treatment samples.

**Table 4:** Instrinsic subset assignments in completers with at least two skin biopsies

Patient	Response	Baseline subset	Post-treatment subset
N5	Improver	Inflammatory	Fibroproliferative
N10	Improver	Fibroproliferative	Normal-like
N11	Improver	Inflammatory	Normal-like
N16	Improver	Normal-like	Normal-like
N15	Non-improver	Fibroproliferative	Fibroproliferative
N19	Non-improver	Fibroproliferative	Fibroproliferative

#### TGFBR and PDGFRB signaling pathways are highly expressed in improvers at baseline

**Fig. 3:**
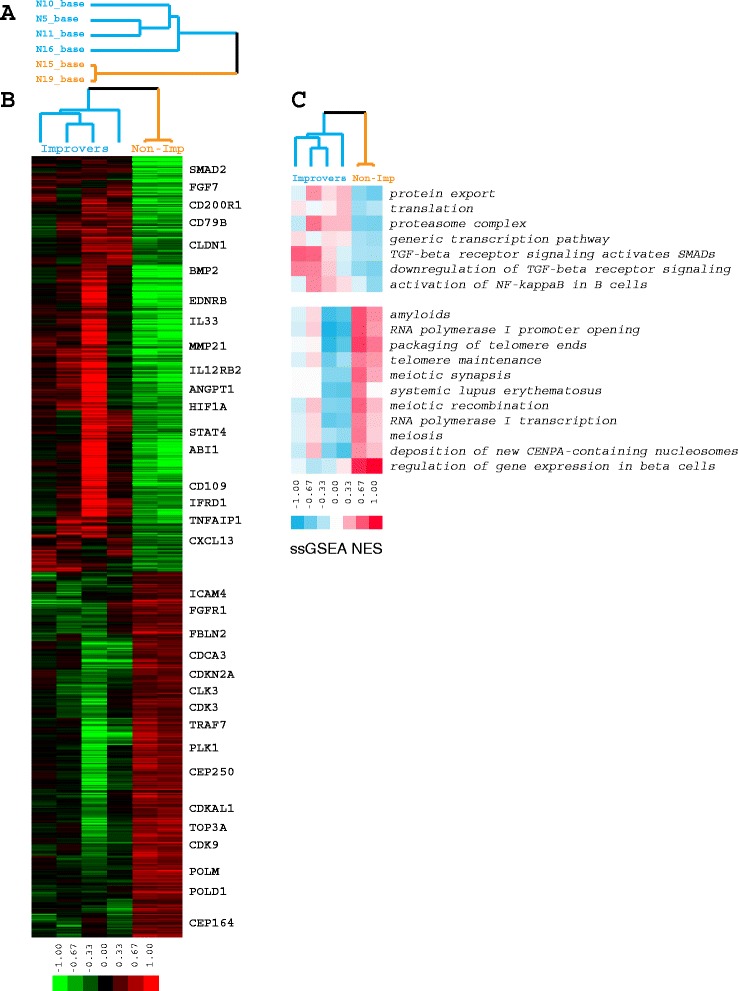
Baseline differential gene expression and pathway enrichment analysis. **a** Baseline sample tree: *blue* improvers, *orange* non-improvers. **b** Sample genes differentially expressed at baseline between improvers and non-improvers. **c** Pathways differentially expressed at baseline between improvers and non-improvers

We performed differential gene expression analysis between baseline improver (n = 4) and non-improver (n = 2) samples (Fig. 3a): 2,242 genes were significantly differentially expressed at baseline (*p* <0.05, unpaired *t* test) (Fig. 3b).

We used GSEA to identify 18 pathways with significantly differential expression between improvers and non-improvers (FDR <5 %; corrected for multiple testing). Pathways that increased in improvers included TGFB receptor signaling (*TGFBR*) and nuclear factor kappa B (*NFKB*) signaling, whereas pathways with increased expression in non-improvers included telomere maintenance and meiosis, suggesting enrichment in terms associated with the cell cycle and cell proliferation, consistent with the assignment of the two non-improvers to the fibroproliferative subset (Fig. 3c).

**Fig. 4:**
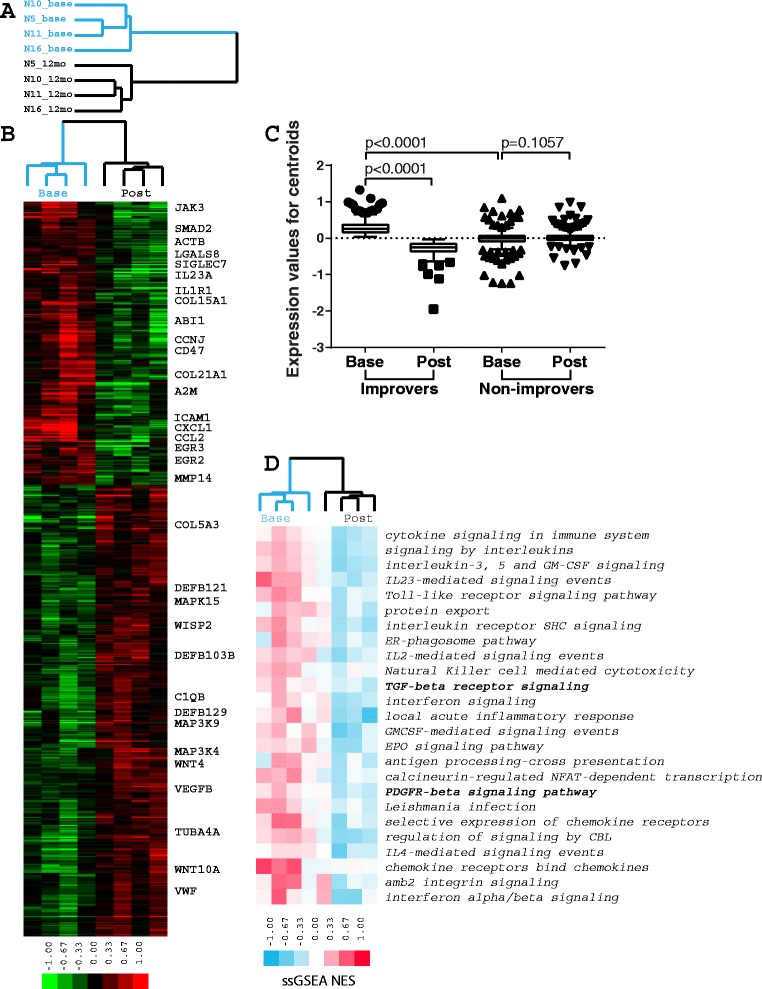
Improver differential gene expression and pathway enrichment analysis. **a** Improver array tree: *blue* baseline samples, *black* post-treatment samples. **b** Sample genes differentially expressed in improvers between baseline and post-treatment biopsies. **c** Improver gene signature trends across improver and non-improver samples. Graphs represent Tukey box and whiskers plots. **d** Pathways differentially expressed in improvers between baseline and post-treatment biopsies

The four improvers with baseline and 12-month biopsies were included in the microarray data analysis (Fig. 4a). We identified 666 genes significantly differentially expressed in improvers between baseline and post treatment (*p* <0.05, paired *t* test) that comprised an improver gene signature (Additional file 5). Genes that showed decreased expression post treatment included *TGFB*-regulated (*SMAD2*, *ACTB*, *COL15A1*, *ABI1*, *EGR2* and *EGR3*) and inflammatory (*SIGLEC7*, *IL23A*, *ICAM1*, *CCL2* and *MMP14*) gene signatures (Fig. 4b, Additional file 5). These genes had significantly higher expression in improvers at baseline relative to non-improvers, and were significantly decreased in improvers post treatment (*p* <0.0001 for both comparisons). In contrast, their expression remained stable in non-improvers (*p* = 0.1057) (Fig. 4c). Non-improvers had 74 genes with significant differential expression between baseline and post-treatment samples, and no significant overlap with the improver signature. There were only 2/74 genes in common with the improver gene signature but their directionality was opposite to that for improvers.

We identified 25 pathways that were highly significantly decreased in improvers post-treatment (FDR <1 %), including multiple immune response pathways such as interleukin and interferon signaling (Fig. 4d). A broader list of 106 pathways that were significantly decreased in improvers post treatment (FDR <5 %) is shown in Additional files 6 and 7. We were interested in *TGFBR* and *PDGFRB* pathways because of their relevance to fibrosis in SSc and their convergence on *c-Abl*, a target of nilotinib. We identified 19 genes involved in *TGFBR* signaling (including *TGFB2*, *TGFB3*, *TGFBR1* and *TGFBR2*) and 50 genes involved in *PDGFRB* signaling (including *PDGFRB* and *PDGFB*) that comprised the core enrichment groups for these pathways in improvers based on GSEA results (Additional file 8).

**Fig. 5:**
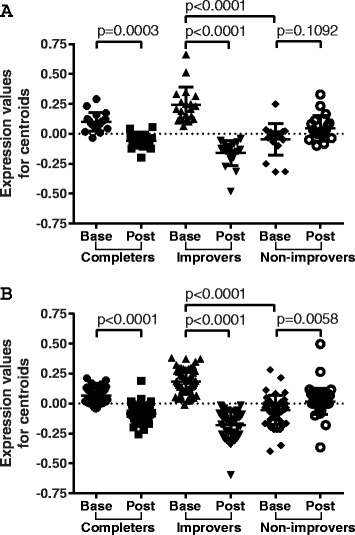
Transforming growth factor beta receptor (*TGFBR*) and platelet-derived growth factor receptor beta (*PDGFRB*) signaling trends across nilotinib patients. **a** Expression trends for TGFBR signaling pathway across completers, improvers and non-improvers. **b** Expression trends for PDGFRB signaling pathway across completers, improvers and non-improvers. Scatter plots show mean with standard deviation

We examined the expression of *TGFBR* and *PDGFRB* signaling gene sets across six completers (combined improvers and non-improvers), four improvers and two non-improvers at baseline and post treatment (Fig. 5). Both pathways were significantly higher at baseline in improvers compared to non-improvers (*p* <0.0001 for both gene sets). Both pathways significantly decreased post treatment in improvers (*p* <0.0001 for both gene sets). While genes associated with *TGFBR* signaling showed stable expression in non-improvers (*p* = 0.1092) (Fig. 5a), *PDGFRB* signaling genes were significantly increased in non-improvers post treatment (*p* = 0.0058; Fig. 5b). No pathways were significantly differentially expressed in samples from non-improvers at baseline and post-treatment.

## Discussion

The initial positive in vitro and murine studies on the use of TKIs with *c-Abl* and *PDGFR* inhibition (e.g., imatinib, nilotinib, and dasatinib) for dcSSc presented the hope for a profound treatment effect for the cutaneous fibrosis seen in dcSSc. Several studies have attempted to address the safety and effectiveness of imatinib in this population, but design limitations make it impossible to make definitive conclusions. The collective imatinib experience has been mixed. A modest improvement in skin thickening was seen in some open-label studies, but side effects, especially fluid retention, were prevalent and prevented some patients from continuing the medication. This paper is the first published report on the use of nilotinib for the treatment of dcSSc.

In this pilot study of nilotinib, a different but related TKI with fewer fluid-related side effects, our goals were to assess safety in a population with dcSSc, generate pilot data on the MRSS to help us adequately power future studies, and to use histopathology and gene expression profiling to further understand our clinical observations.

Nilotinib was well-tolerated by the majority of patients in this study. Although the number of AE that were at least possibly related to nilotinib was high, many of the AE represented asymptomatic laboratory abnormalities that resolved without intervention. The side effects observed were similar to what has been seen in patients with CML [30, 31]. However, nilotinib is known to have important cardiovascular side effects, which were observed and are particularly relevant to SSc patients. Monitoring the QTc with periodic EKG is required. Conduction abnormalities including QTc prolongation can be seen in dcSSc patients [32] or as a side effect of other medications they may use. In our study QTc prolongation was seen in five SSc patients at screening, which limited their eligibility. Persistent mild QTc prolongation without arrhythmia was the cause of two patients discontinuing treatment. Acceleration of peripheral arterial vascular disease has been reported with nilotinib in CML patients both with and without other risk factors for CAD [33, 34]. In this small study we observed exacerbation of preexisting CAD in one patient with other risk factors. Careful consideration of the use of this medicine in patients with CAD or multiple risk factors would be needed in patients with SSc, as it would in CML.

We observed that the MRSS improved in a statistically significant manner with 6 and 12 months of treatment in this group of patients with very early and active SSc. The range of the minimally clinically important difference in MRSS has been estimated to be 3.2−5.3 points [35]. Therefore, the patients in this study were observed to change in a clinically meaningful manner. At the time of study entry the median disease duration was 0.7 years, 70 % of patients had a worsening MRSS, 50 % had the RNA Polymerase3 antibody and 40 % had tendon friction rubs. The combination of these clinical attributes put these patients at high risk of exacerbation of disease during the study period. However, the MRSS improvement is inconclusive given the uncontrolled study design and the observation that the MRSS tends to improve both with time [36] and in the context of clinical trials [37]. Nonetheless, this clinical finding of improved MRSS in group of patients with such early and active dcSSc is worthy of further and more definitive study.

The FVC and DLCO were not significantly changed, but our trial was not designed to evaluate pulmonary endpoints, and most patients in this study did not have ILD. Recently another TKI, nintedanib, which has some overlapping spectrum of TK inhibition, has been shown to slow the decline of FVC in idiopathic pulmonary fibrosis, a related fibrotic condition [38]. The additional outcome measures, including the SF-36 and SHAQ, at the 6- and 12-month time points also remained stable, and this has been shown to occur typically in SSc clinical trials [39].

In this study we used gene expression profiling and the concept of SSc intrinsic subsets to characterize biological response and distinguish between nilotinib improvers and non-improvers. In terms of the intrinsic subset assignment, three of four improvers were classified as non-fibroproliferative (with two out of four classified as inflammatory), whereas non-improvers were classified as fibroproliferative at baseline. Improvers tended to lose their intrinsic subset signature and become normal-like post treatment, while non-improvers remained fibroproliferative (Table 4). Genes from specific pathways targeted by nilotinib (e.g., *TGFBR* and *PDGFRB* signaling) showed significantly higher expression in improvers at baseline and were significantly downregulated in those improvers by treatment, whereas their expression in non-improvers was stable or displayed directionality opposite to improvers, with low expression at baseline. Interestingly, a recent analysis of imatinib and nilotinib effects in SSc mouse models suggested that high activation status and expression pattern of TKI targets was predictive of the potential response to therapy [8]. It is not clear based on this single-group and uncontrolled clinical trial whether our results represent the natural history of the disease or a treatment effect, although an apparent change in the post-treatment intrinsic subset assignment to the one with less severe disease in improvers might reflect beneficial effects of the therapy. As the use of gene expression in the context of clinical trials continues, the significance of these findings will be more conclusive.

In this study, we found that *TGFBR* signaling was significantly increased in improvers at baseline and that this signature spanned the inflammatory and fibroproliferative subsets. In our original study of *TGFB* signaling in SSc skin [40] we observed its enrichment primarily in the fibroproliferative subset. However, a recent meta-analysis of three independent gene expression datasets from SSc skin, which dramatically increases the number of samples analyzed, shows that *TGFBR* signaling appears to span the inflammatory and fibroproliferative subsets [41, 42].

The results here are consistent with those data, as our improvers, who are primarily defined by high expression of *TGFBR* signaling at the initiation of treatment, appear to span across the fibroproliferative and inflammatory intrinsic gene expression subsets. This indicates that a biomarker of *TGFBR* signaling will be a useful stratification method in addition to biomarkers of the intrinsic gene expression subsets.

The histopathology of the forearm skin biopsy did not show a significant morphological improvement even in cases where a clinical improvement was seen, and phospho-staining of known targets of nilotinib in the skin was not differentially activated before and after treatment. It is possible that these dermatopathological investigations are less sensitive than microarray in picking up this degree of change, and this would be a topic for future investigation.

There were several limitations to this study, including its open-label design and small sample size. Because of these factors conclusions about efficacy cannot be definitively drawn. As noted, the MRSS tends to improve due to regression to the mean [35] and in the context of clinical trials [36]. The lack of a control group additionally makes the changes in gene expression observed in the microarray studies inconclusive with respect to whether they represent a change due to treatment or a change based on the natural history of the disease. In terms of the differential gene expression analyses, we had to use uncorrected *p* values in the unpaired and paired *t* tests as the small sample size precluded the adjustment for multiple comparisons. We controlled for this by focusing on pathways found to be deregulated using GSEA, which were corrected for multiple hypothesis testing. This was a pilot study designed to provide preliminary data that could drive more conclusive hypothesis testing.

## Conclusions

This was an open-label, single-group pilot trial, which included histopathologic and microarray analyses of skin to further delineate biological response. In this pilot clinical trial a significant improvement in the MRSS was observed in patients who would have been expected to have progression of disease. Seventy percent of patients were able to tolerate the medication for 12 months, and side effects were predictable. Improvers were most clearly defined by having high expression of genes associated with *TGFBR/PDGFRB* signaling at the time of treatment initiation, while those that did not improve did not have evidence of activation. *TGFBR* and *PDGFRB* signaling pathways significantly decreased in improvers post treatment and this was not observed in non-improvers. Further evaluation of nilotinib or related TKIs in a randomized and controlled setting is warranted.

## Additional files

Additional file 1:
**All adverse events.** This table lists all adverse events recorded in order of frequency. Grades listed are based on definitions set forth in CTCAE v2.0. Attribution as per the investigators and depending on the clinical scenario is listed as well. 1 signifies not related to the medication, 2 signifies unlikely related to the medication, 3 signifies possibly related to the medication, 4 signifies probably related to the medication, and 5 signifies definitely related to the medication. Some side effects have different attribution for different events given clinical context. *QTC* corrected QT interval, *ESR* erythrocyte sedimentation rate, *CABG* coronary artery bypass graft. (DOCX 17 kb) Additional file 2:
**Dermatopathology results.** *αSMA* alpha smooth muscle actin, *Egr* early growth response protein. (DOCX 15 kb) Additional file 3:
**List of 651 intrinsic genes used to cluster nilotinib samples in Fig.** **2** **.** Gene symbols are color-coded based on their association with inflammatory (*purple*), normal-like (*green*) or fibroproliferative (*red*) intrinsic subsets. (XLSX 32 kb) Additional file 4:
**Spearman correlation statistics (correlation coefficients and** ***p*** **values) for intrinsic subset assignment.** (XLSX 11 kb) Additional file 5:
**Detailed output for the 666 gene signature in improvers from Fig.** **4b** **.** For each gene in the improver gene signature, the following information is provided: where it is upregulated (baseline or post treatment), gene symbol, gene name, raw *p* value, false discovery rate (FDR)(BH), *q* value, fold change, baseline mean expression value and post-treatment mean expression value. (XLSX 73 kb) Additional file 6:
**List of 106 pathways significantly downregulated in improvers post treatment (false discovery rate (FDR) <5 %).** The FDR is provided for each pathway. (XLSX 13 kb) Additional file 7:
**Entire output for the 106 pathway signature in improvers from gene set enrichment analysis (GSEA).** (PDF 367 kb) Additional file 8:
**Heat maps for core enrichment genes from transforming growth factor beta receptor (TGFBR) (** **A** **) and platelet-derived growth factor receptor beta (PDGFRB) (** **B** **) signaling pathways corresponding to Fig.** **5** **.** (PDF 316 kb)

## Abbreviations

ABI1: abl-interactor 1
ACTB: actin beta
AE: adverse event
cAbl: Abelson murine leukemia viral oncogene homolog
CAD: coronary artery disease
CCL2: chemokine (C-C motif) ligand 2
CML: chronic myelogenous leukemia
COL15A1: collagen 15a1
dcSSc: diffuse cutaneous systemic sclerosis
DLCO: diffusion lung capacity of carbon monoxide
EF: ejection fraction
EGR: early growth response protein
EKG: electrocardiogram
ESR: erythrocyte sedimentation rate
FDR: false discovery rate
FVC: forced vital capacity
GSEA: gene set enrichment analysis
H&E: hematoxylin and eosin
ICAM1: intercellular adhesion molecule 1
IL23A: interleukin 23A
ILD: interstitial lung disease
lcSSc: limited cutaneous systemic sclerosis
MMP14: matrix metalloproteinase 14
MRSS: modified Rodnan skin score
NFKB: nuclear factor kappa-light-chain-enhancer of activated B cells
PCL: preclustering
PDGF: platelet-derived growth factor
PDGFR: platelet-derived growth factor receptor
PFT: pulmonary function test
PGA: physician global assessment
proCol: procollagen
QTc: corrected QT interval - referring to an interval on an electrocardiogram
SAE: serious adverse event
SF-36: short form 36-item health survey
SF-36 MC: short form 36-item health survey mental component
SF-36 PC: short form 36-item health survey physical component
SHAQ-DI: scleroderma health assessment questionnaire - disability index
SIGLEC7: sialic acid-binding immunoglobulin-type lectin 7
SSc: systemic sclerosis
TC: trichrome
TGFB: transforming growth factor beta
TGFBR: transforming growth factor beta receptor
TKI: tyrosine kinase inhibitor
αSMA: alpha smooth muscle actin
