# Supplementary material for: Nilotinib (Tasigna™) in the treatment of early diffuse systemic sclerosis: an open-label, pilot clinical trial
Source: Arthritis Res Ther. 2015 Aug 18;17(1):213. doi: 10.1186/s13075-015-0721-3 (PMC4538758; doi:10.1186/s13075-015-0721-3)
Supplement: Additional file 1: — All adverse events. This table lists all adverse events recorded in order of frequency. Grades listed are based on definitions set forth in CTCAE v2.0. Attribution as per the investigators and depending on the clinical scenario is listed as well. 1 signifies not related to the medication, 2 signifies unlikely related to the medication, 3 signifies possibly related to the medication, 4 signifies probably related to the medication, and 5 signifies definitely related to the medication. Some side effects have different attribution for different events given clinical context. QTC corrected QT interval, ESR erythrocyte sedimentation rate, CABG coronary artery bypass graft. (DOCX 17 kb) [file 13075_2015_721_MOESM1_ESM.docx]

Table S1. All Adverse Events

| Adverse Event | Frequency of Event | # of Patients Reporting | % of Patients Reporting | Grade (n) | Attribution (n) |
| --- | --- | --- | --- | --- | --- |
| Prolonged QTC | 9 | 6 | 86 | 1 (8), 2 (1) | 4 |
| Increased AST | 6 | 5 | 71 | 1 | 3 (3), 4 (3) |
| Elevated Total Bilirubin | 6 | 5 | 71 | 1 | 3 (3), 4 (3) |
| Increased ALT | 5 | 4 | 57 | 1 | 3 (2), 4 (3) |
| Anemia | 3 | 3 | 43 | 1,2,3 | 3 |
| Hyperglycemia | 3 | 3 | 43 | 1 | 3 |
| Headache | 3 | 3 | 43 | 1 | 3,4 |
| Nausea | 2 | 2 | 29 | 1 | 3,4 |
| Reflux | 2 | 2 | 29 | 1,2 | 2,3 |
| Elevated ESR | 2 | 2 | 29 | 1 | 1,2 |
| Increased Lipase | 2 | 2 | 29 | 2,3 | 3,4 |
| Increased Amylase | 2 | 2 | 29 | 1 | 3,4 |
| Upper Respiratory Infection | 2 | 2 | 29 | 1 | 1,2 |
| Vomiting | 2 | 1 | 14 | 1 | 1 |
| Decreased WBC Count | 1 | 1 | 14 | 1 | 3 |
| Increased WBC Count | 1 | 1 | 14 | 1 | 2 |
| Coronary Artery Disease | 1 | 1 | 14 | 3 | 3 |
| Syncope | 1 | 1 | 14 | 4 | 3 |
| Hospitalization For CABG | 1 | 1 | 14 | 4 | 3 |
| Allergic Rhinitis | 1 | 1 | 14 | 2 | 1 |
| Cataracts | 1 | 1 | 14 | 3 | 2 |
| Conjuctivitis | 1 | 1 | 14 | 1 | 1 |
| Eye Disorder | 1 | 1 | 14 | 2 | 1 |
| Diarrhea | 1 | 1 | 14 | 1 | 4 |
| Edema | 1 | 1 | 14 | 1 | 1 |
| Interim Bacterial Sinusitis | 1 | 1 | 14 | 2 | 1 |
| Skin Infection | 1 | 1 | 14 | 2 | 2 |
| Low Inorganic Phosphorus | 1 | 1 | 14 | 1 | 4 |
| Vitamin D Deficiency | 1 | 1 | 14 | 2 | 3 |
| Ankle Arthritis | 1 | 1 | 14 | 2 | 2 |
| Plantar Fascitis | 1 | 1 | 14 | 1 | 2 |
| Calcinosis | 1 | 1 | 14 | 1 | 1 |
| Dizziness | 1 | 1 | 14 | 2 | 3 |
| Concentration Impairment | 1 | 1 | 14 | 1 | 3 |
| Anxiety | 1 | 1 | 14 | 2 | 3 |
| Erectile Dysfunction | 1 | 1 | 14 | 2 | 3 |
| Rash | 1 | 1 | 14 | 2 | 5 |
| Alopecia | 1 | 1 | 14 | 1 | 3 |
