# Supplementary material for: Nilotinib (Tasigna™) in the treatment of early diffuse systemic sclerosis: an open-label, pilot clinical trial
Source: Arthritis Res Ther. 2015 Aug 18;17(1):213. doi: 10.1186/s13075-015-0721-3 (PMC4538758; doi:10.1186/s13075-015-0721-3)
Supplement: Additional file 2: — Dermatopathology results. αSMA alpha smooth muscle actin, Egr early growth response protein. (DOCX 15 kb) [file 13075_2015_721_MOESM2_ESM.docx]

| Measurement | Baseline (n=8) | 6 month (n=8) | 12 month (n=6) | P-value 6 month | p-value 12 month |
| --- | --- | --- | --- | --- | --- |
| Thickness (mm, mean ± SD) | 2.30 ± 0.6 | 2.75 ± 0.58 | 2.63 ± 0.32 | 0.0639 | 0.3566 |
| Infiltrate (score, mean ± SD) | 0.75  ± 0.27 | 0.63  ± 0.44 | 0.58  ±0.2 | 0.5165 | 0.3632 |
| Collagen density (score, mean ± SD) | 1.75  ±0.84 | 2.3  ± 0.75 | 2.08  ± 0.66 | 0.2764 | 0.6462 |
| Eccrine and follicular structures (number, mean ± SD) | 1.75  ± 1.28 | 2.75  ± 1.28 | 2.17  ± 0.75 | 0.08561 | 0.4650 |
| α-SMA stain (score, mean ± SD) | 0.69  ± 0.75 | 0.63  ± 0.79 | 0.75  ± 0.93 | 0.8436 | 0.8892 |
| Masson Trichrome stain (score, mean ± SD) | 1.69  ±0.88 | 2.31  ±0.75 | 2.17  ± 0.75 | 0.2417 | 0.5301 |
| CD34 stain (score, mean ± SD) | 1.56  ± 0.73 | 1.44  ± 0.86 | 2.0  ± 0.63 | 0.7799 | 0.4150 |
| ProCollagen stain (score, mean ± SD) | 1.25  ± 1 | 1.94  ± 0.86 | 1.58  ± 0.92 | 0.1204 | 0.7184 |
| Egr stain (score, mean ± SD) | 1.5  ± 0.46 | 1.75  ± 0.65 | 1.83  ± 0.68 | 0.3506 | 0.3632 |

|  |  |
| --- | --- |
