# Supplementary figures and images for: Nilotinib (Tasigna™) in the treatment of early diffuse systemic sclerosis: an open-label, pilot clinical trial
Source: Arthritis Res Ther. 2015 Aug 18;17(1):213. doi: 10.1186/s13075-015-0721-3 (PMC4538758; doi:10.1186/s13075-015-0721-3)

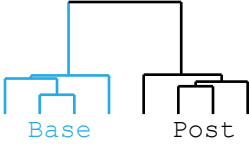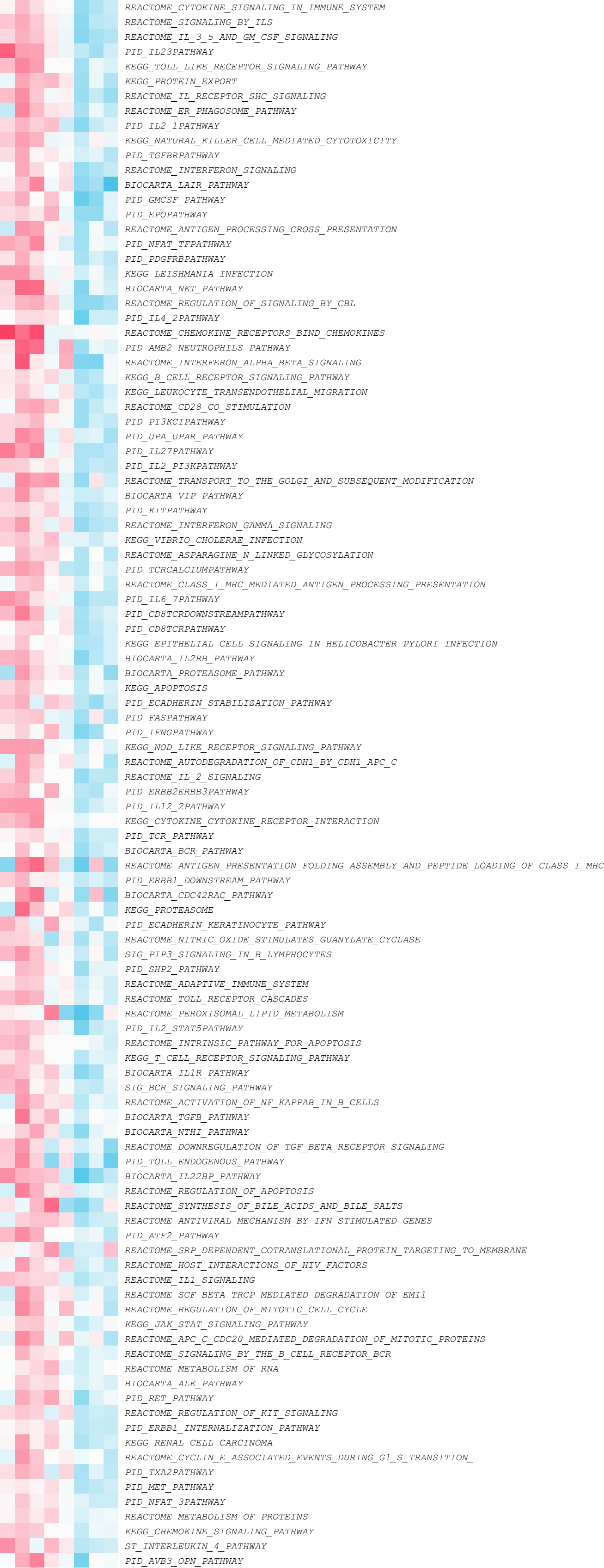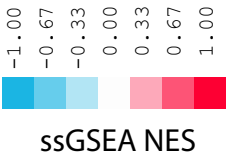

Supplement: Additional file 7: — Entire output for the 106 pathway signature in improvers from gene set enrichment analysis (GSEA). (PDF 367 kb) [file 13075_2015_721_MOESM7_ESM.pdf]

**A**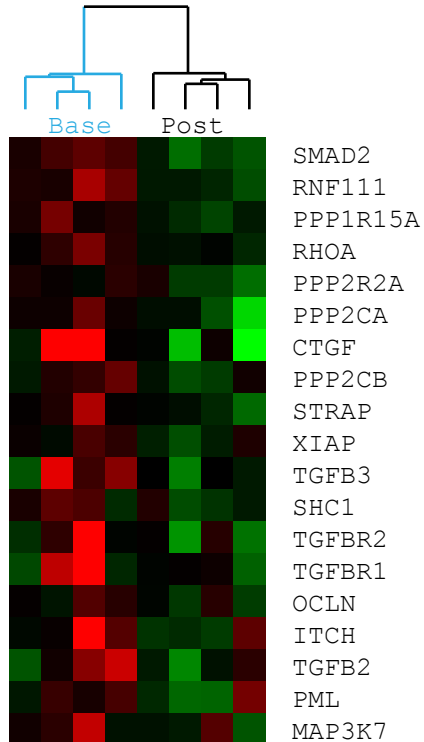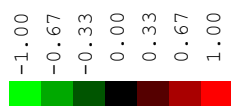**B**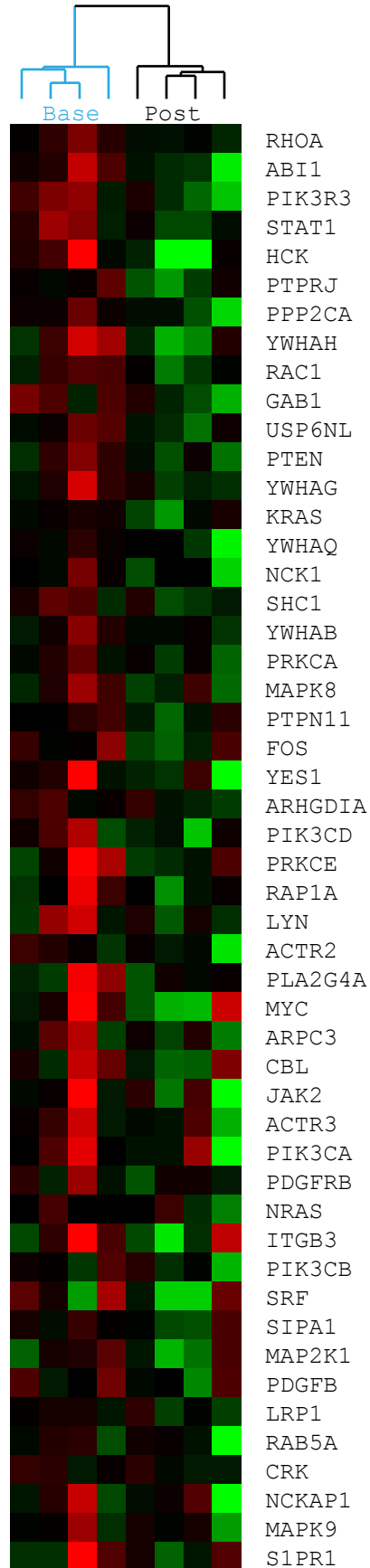

Supplement: Additional file 8: — Heat maps for core enrichment genes from transforming growth factor beta receptor (TGFBR) ( A ) and platelet-derived growth factor receptor beta (PDGFRB) ( B ) signaling pathways corresponding to Fig. 5 . (PDF 316 kb) [file 13075_2015_721_MOESM8_ESM.pdf]
